# ﻿New Philopotamidae (Insecta, Trichoptera) from Ecuador: seven new species and updated country checklist

**DOI:** 10.3897/zookeys.1263.147996

**Published:** 2025-12-10

**Authors:** Ralph W. Holzenthal, Roger J. Blanhik, Blanca Ríos-Touma

**Affiliations:** 1 Department of Entomology, University of Minnesota, 1980 Folwell Avenue, 219 Hodson Hall, St. Paul, Minnesota 55108, USA University of Minnesota St. Paul United States of America; 2 Facultad de Ingenierías y Ciencias Aplicadas, Ingeniería Ambiental, Grupo de Investigación en Biodiversidad Medio Ambiente y Salud –BIOMAS- Universidad de Las Américas, Quito, Ecuador Universidad de Las Américas Quito Ecuador

**Keywords:** Aquatic insects, caddisflies, *
Chimarra
*, *
Chimarrhodella
*, *
Sumacodella
*, taxo­nomy, *
Wormaldia
*

## Abstract

Seven new species in the caddisfly family Philopotamidae are diagnosed, described, and illustrated from Ecuador, three in the subfamily Chimarrinae: *Chimarrhodella spinosa***sp. nov.**, Chimarra (Otarrha) buglas**sp. nov**., Chimarra (Chimarra) buenaventura**sp. nov.**, and four in the subfamily Philopotaminae: *Sumacodella grijalvai***sp. nov.**, *Wormaldia nilssoni***sp. nov.**, *Wormaldia insolita***sp. nov.**, and *Wormaldia milpe***sp. nov.** The species are rare and endemic to the country. In addition, an updated distributional checklist of the 60 species of philopotamids now known from the country is presented. Thirty-eight, or 64%, of these species are endemic. Finally, the Chao 2 estimator, based on 126 localities, suggests that the expected number of philopotamid species is 60 (SD 9), which implies that almost all the species present in the country are known.

## ﻿Introduction

The cosmopolitan family Philopotamidae is highly diverse within tropical regions, where numerous novel species have been described (e.g., Holzenthal et al. 2018). The family encompasses 24 genera distributed among three subfamilies: Chimarrinae, Philopotaminae, and Rossodinae (Blahnik 2005). Seven philopotamid genera occur in the Neotropics, including the widely distributed genera *Wormaldia* and *Chimarra*, recognized as one of the most species rich genera within the order (Kjer et al. 2014), but also endemic genera such as *Sumacodella*, known previously from a single species from Ecuador (Holzenthal et al. 2022), *Alterosa*, *Aymaradella*, *Chimarrhodella*, and *Sortosa*. Neotropical regions, particularly the Tropical Andes encompassing Ecuador and adjacent countries, represent significant reservoirs of largely unexplored Trichoptera diversity (Ríos-Touma et al. 2017, 2022). Despite taxonomic descriptions of more than 3000 Trichoptera species within the Neotropics (Holzenthal and Calor 2017), dozens of species continue to be described every year. The Tropical Andean region, especially, encompasses numerous underexplored aquatic habitats, thus suggesting that the number of known species is a significant underestimate of the true diversity.

We have been implementing intensive efforts in the last five years to increase our knowledge of the diversity of Ecuadorian Trichoptera. Here, we describe seven new species of Philopotamidae: one *Chimarrodella*, two *Chimarra*, one species in the endemic genus *Sumacodella*, and three new *Wormaldia* species. Additionally, we provide an updated list of the Philopotamidae of Ecuador.

## ﻿Materials and methods

Adult specimens were collected at ultraviolet LED lights placed adjacent to streams. The lights were put on a white tray with ethanol or hung in front of a white bed sheet connected to a USB power pack and left for 2 hours after dusk.

Adult specimens underwent standard preparation and examination procedures for pinned or alcohol-preserved material (Blahnik and Holzenthal 2004; Blahnik et al. 2007). Forewing length was measured from base to apex. Male genitalia were subjected to immersion in 85% lactic acid at 125 °C for 30–60 minutes to dissolve internal soft tissues. Utilizing an Olympus BX41 compound microscope equipped with a drawing tube, specimens were observed, and detailed pencil drawings of genitalic structures were produced. These sketches were digitally scanned and imported into Adobe Illustrator to create vector illustrations. Morphological terminology adhered to that outlined by Blahnik (1998) and Holzenthal et al. (2022). Each specimen was assigned a matrix barcode label featuring a unique alphanumeric sequence commencing with the prefix UMSP, serving as a distinct identifier for specimen data uploaded to the University of Minnesota Insect Collection (UMSP) Specify (specifysoftware.org) database and is available through the Global Biodiversity Information Facility GBIF (gbif.org), including latitude, longitude, elevation and distribution maps for each species.

Types of the new species and other material examined are deposited in the University of Minnesota Insect Collection, St. Paul, Minnesota, USA (**UMSP**) and the Museo Ecuatoriano de Ciencias Naturales, Instituto Nacional de Biodiversidad, Quito, Ecuador (**MECN**). Type specimens with only one individual are temporally deposited in UMSP.

Including the new species described here and those previously recorded or described from Ecuador (Holzenthal and Calor 2017; Holzenthal et al. 2018, 2022), we provide an updated list of the described species of Ecuadorian Philopotamidae. With our collection data, based on 126 collecting sites where we identified male Philopotamidae, we calculated the Chao 2 species estimator, which uses incidence data to estimate the true diversity. This is highly sensitive to singletons (species presence in only one locality) (Chao et al. 2009). We used only our collecting sites because they were surveyed similarly. Therefore, the results are comparable. We calculated this estimator using Primer 7 stati­stical software (Clarke and Gorley 2015).

## ﻿Results

### ﻿Systematics

**Chimarrinae**

#### ﻿*Chimarrhodella*

*Chimarrhodella* is a relatively small genus confined to the Neotropics, significant in being the sister taxon to the very large and cosmopolitan genus *Chimarra*. The genus was revised by Blahnik and Holzenthal (1992a), and several additional species were subsequently added by Blahnik (2004) and Holzenthal et al. (2018). The genus currently contains thirteen species, including *Chimarrhodella aequatoria* (Navás), described from Ecuador from an unidentifiable female specimen, now missing. This new species adds a fourteenth species to the genus and increases the number of species known from Ecuador to six (or five identifiable ones).

##### ﻿ Chimarrhodella spinosa sp. nov.

Taxon classificationAnimaliaTrichopteraPhilopotamidae

94A55B88-750A-5D0D-A919-D64B2F580413

https://zoobank.org/533CC891-65D5-4DA3-AF8C-A30A0C8B97C2

Fig. 1

###### Type material.

***Holotype*** male: **Ecuador**: • Zamora Chinchipe: Tributary crossing Hwy E682, N of Valladolid, Tapichalaca Reserve, 4.53371°S, 79.13041°W, el. 1898 m, 12.ii.2023, Ríos, Amigo, Huisman (UMSP000279961) (MECN).

###### Diagnosis.

*Chimarrhodella spinosa* is very closely related to *C. costaricensis* Blahnik & Holzenthal, 1992, resembling it particularly in the structure of tergum X, which is membranous mesally and has sensillate lateral projections, each rounded apically, with the apex reflexed on its ventral margin to form a prominent, acute, ventrally projecting, spine-like projection at approximately midlength. Also, like *C. costaricensis*, and unlike other species in the *galeata* group of *Chimarrhodella*, it has the apices of the inferior appendage acute, resembling species of the *peruviana* group in this respect. *Chimarrhodella spinosa* is most readily distinguished from *C. costaricensis* by the spines of the phallic apparatus. Like *C. costaricensis*, it has a pair of short, symmetrically positioned, phallic spines basally, although slightly longer in length than in *C. costaricensis*; it differs in having an additional pair of spinose tracts, composed of clustered, fine, needle-like spines, anterior to the paired phallic spines. These tracts of fine spines are a unique feature among known species of the genus. *Chimarrhodella spinosa* also differs from *C. costaricensis* in its somewhat larger size and longer inferior appendage, with their apicomesal margins more abruptly narrowed at approximately midlength.

###### Description.

Forewing length male 7.0 mm. Color pale stramineous, denuded (specimen in alcohol). **Male.** Segment IX, in lateral view, with anteroventral margin weakly produced, concavely excised mesally; posterolateral margins with weakly developed, setose, dorsolateral expansions at level of preanal appendage. Tergum X membranous mesally, shallowly notched at apex, laterally with pair of sclerotized, apically sensillate lobes; each lobe with large sclerotized, spine-like, anteroventrally directed projection. Preanal appendage elongate, but short in comparison to other species of *Chimarrhodella*; inserted dorsad of widest point of posterolateral expansion of segment IX. Inferior appendage, in lateral view, elongate, linear; in ventral view, widest basally, narrowing apically, abruptly and concavely narrowed on mesal surface in apical half, apex with short, acute, mesally directed projection. Phallus with phallobase tubular, elongate, expanded basodorsally, extended posteroventrally; endotheca elongate, with well-developed, preapical, pleated, hood-like expansion; basally with pair of subequal, elongate, phallic spines, lacking basal enlargements, and additional paired tracts of fine, needle-like spines; phallotremal sclerite complex elongate, tube-like, weakly sclerotized overall, slightly more sclerotized basally; ventral margin with wide sclerotized strip, narrowing preapically, weakly forked and more sclerotized apically.

**Figure 1.**
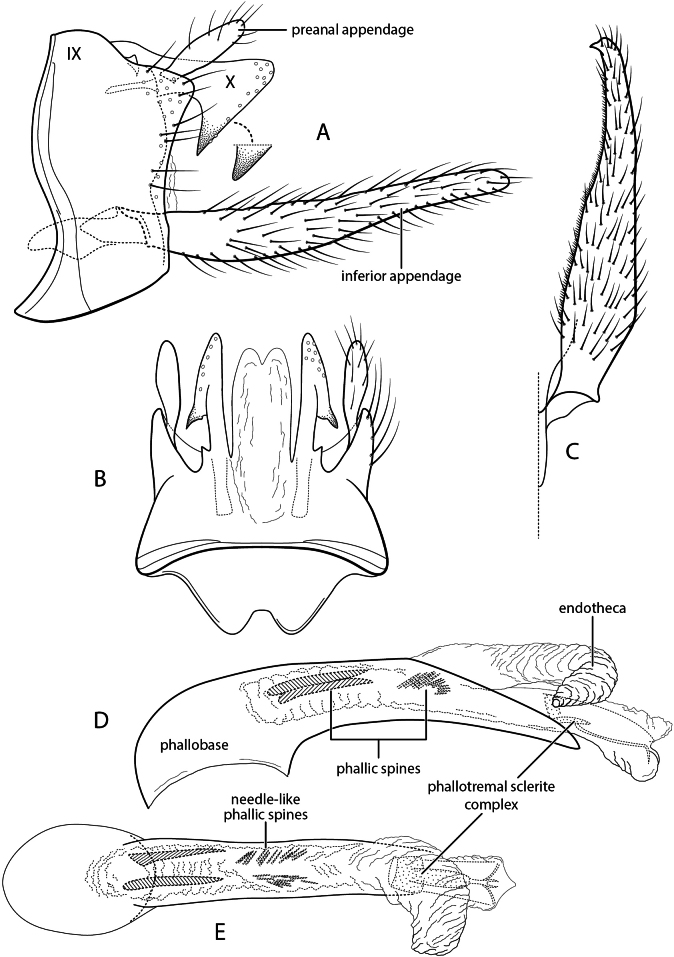
*Chimarrhodella spinosa* new species. Male genitalia **A** segments IX–X, lateral (inset: apex of segment X, right side) **B** segments IX–X, dorsal **C** inferior appendage, ventral **D** phallus, lateral **E** phallus, dorsal.

**Female.** Unknown.

###### Etymology.

The name refers to the tract of fine spines in the endotheca, a unique feature among known species of the genus.

#### ﻿*Chimarra*

##### ﻿ Chimarra (Otarrha) buglas sp. nov.

Taxon classificationAnimaliaTrichopteraPhilopotamidae

AFA0645D-3950-5941-BD63-82F0CA638087

https://zoobank.org/42250F24-AC2F-48C8-B3FC-55FE75C87C1A

Fig. 2

###### Type material.

***Holotype*** male: **Ecuador**: • Morona Santiago: tributary to Río San Antonio, between Limón Indanza & San Antonio, 2.99616°S, 78.43163°W, el. 1016 m, 15.ii.2023, Ríos, Holzenthal, Amigo, Huisman (UMSP000551083) (MECN).

###### Diagnosis.

Character similarities between *C. buglas* and *C. ramosa* Holzenthal, Blahnik & Ríos-Touma, 2022 include the general structure of the inferior appendage, with acute mesal projections both basally and at midlength; short, but distinct, projections from the posterior margin of tergum VIII; and an apically truncate posteroventral process on segment IX. The most distinctive and diagnostic feature of *C. buglas* is the structure of tergum X, which like *C. ramosa* is divided mesally, with each lateral lobe also divided into a dorsal and ventral lobe; the ventral lobe, in *C. buglas* is submembranous and weakly sclerotized preapically, ending in a distinctly sclerotized spine-like projection. This is perhaps the most distinctive and diagnostic feature of the species. Other diagnostic differences include the structure of the dorsal lobes of tergum VIII, which are acute apically, rather than rounded, and covered with small spines. Additionally, the ventral process of segment IX, in lateral view, is wider apically, thus more dramatically truncate, and the inferior appendage, as viewed ventrally, has an apex that is more elongate, acute, and mesally curved. The apex of the inferior appendage also lacks a preapical projection.

###### Description.

**Adult.** Forewing length male 4.5 mm. Color pale brown, denuded (specimen in alcohol). **Male.** Tergum VIII with pair of sclerotized, digitate projections from posterior margin, longer than tergum, apices acute, covered with small spines, denser and more elongate apically. Segment IX, in lateral view, with anterior margin nearly straight (slightly expanded in ventral half), segment longest ventrally, just above ventral process, posterior margin sinuously expanded below preanal appendage; posteroventral process elongate (length ~ 2× width), widening apically, apex distinctly truncate. Posteromesal projection of tergum IX short, subtriangular, wide basally, concavely narrowed laterally, acute apically, ~ 1/3 length of lateral lobes of tergum X. Tergum X divided mesally, forming two sclerotized lateral lobes; in lateral view, lateral lobes each also divided into dorsal and ventral lobes; dorsolateral lobe distinctly shorter than ventrolateral lobe, nearly straight, uniform in width, with multiple sensilla apically and short setae dorsally at approximately midlength; ventral lobe, in lateral view, straight, narrow, submembranous preapically, apex laterally curved, strongly sclerotized, spine-like. Preanal appendage large, flattened, ear-like. Inferior appendage elongate, narrow, mesally curved, tapering apically; in ventral view, with apex strongly inturned, tapering, acute; mesal surface with two tine-like projections: basal tine short and acute, median tine elongate, narrow, sinuate, subequal in length to incurved apex of appendage. Phallus with phallobase short, tubular, distinctly ventrally flexed on ventral margin; endotheca expanded apically, with short membranous apicodorsal projection, internally with elongate spine, wide basally, very narrow and acute apically; phallotremal sclerite complex indistinct, bowed, with converging narrow sclerites.

**Figure 2.**
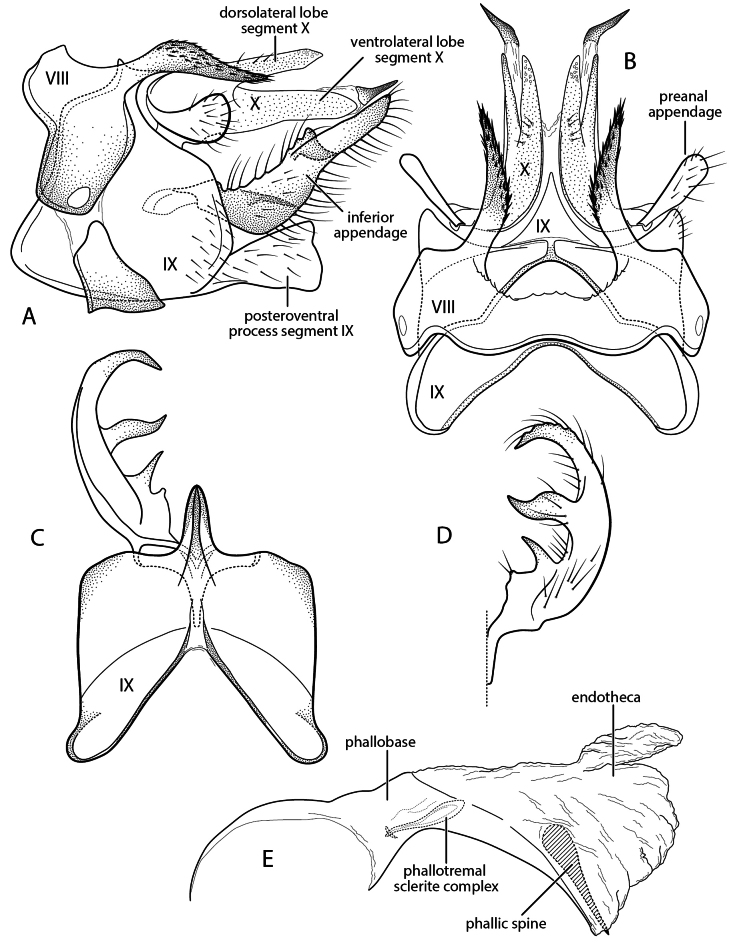
Chimarra (Otarrha) buglas new species. Male genitalia **A** segments VIII–X, lateral **B** segments VIII–X, dorsal **C** inferior appendage, segment IX, ventral **D** inferior appendage, dorsal **E** phallus, lateral.

**Female.** Unknown.

###### Etymology.

Named after the Buglas Nature Reserve, an important area for avian conservation, near the type locality.

###### Remark.

*Chimarra buglas* is a new species in the Chimarra (Otarrha) patosa group, as defined by Blahnik (2002), and likely most closely related to *C. ramosa*. Including the new species, the group now contains eight species. This is the second species described from Ecuador.

##### ﻿ Chimarra (Chimarra) buenaventura sp. nov.

Taxon classificationAnimaliaTrichopteraPhilopotamidae

8B1967F9-F058-58A2-B02B-034DB946FE69

https://zoobank.org/51BA04C5-FF2C-4B2C-A5EA-126D2CF46F4A

Fig. 3

###### Type material.

***Holotype*** male: **Ecuador**: • El Oro: Reserva Buenaventura, small stream near Umbrellabird Lodge, 3.65367°S, 79.76829°W, el. 530 m, 11.x.2023, Ríos-Touma (UMSP000551868) (UMSP). ***Paratypes***: same as holotype, 2 females (MECN); 1 female (UMSP).

###### Diagnosis.

*Chimarra buenaventura* is a particularly distinctive species in the form of its tergum X, with very short and strongly sclerotized lateral lobes, terminating in a distinctive short, narrow projecting sclerite, and with the two sensilla of the lobe located on a recurved process near its distal margin. The phallotremal sclerite of various species in the group is often associated with a textured sclerotized “curl,” modified into a row of short spines in some species. In *Chimarra buenaventura* this structure seems to be modified into a distinct short, curved spine, buttressed basally by a sclerotized projection, appearing as a second short, branched tine. Females of many of the species have been illustrated. The female of *C. buenaventura* is distinguished by a pair of distinctively formed cup-shaped sclerites at the apicoventral attachment site of the vaginal apparatus, and by sinuate, paired sclerites on its ventral surface, which extend anterolaterally to the anterior cup-like sclerite, a generalized feature of the vaginal apparatus in *Chimarra*.

**Figure 3.**
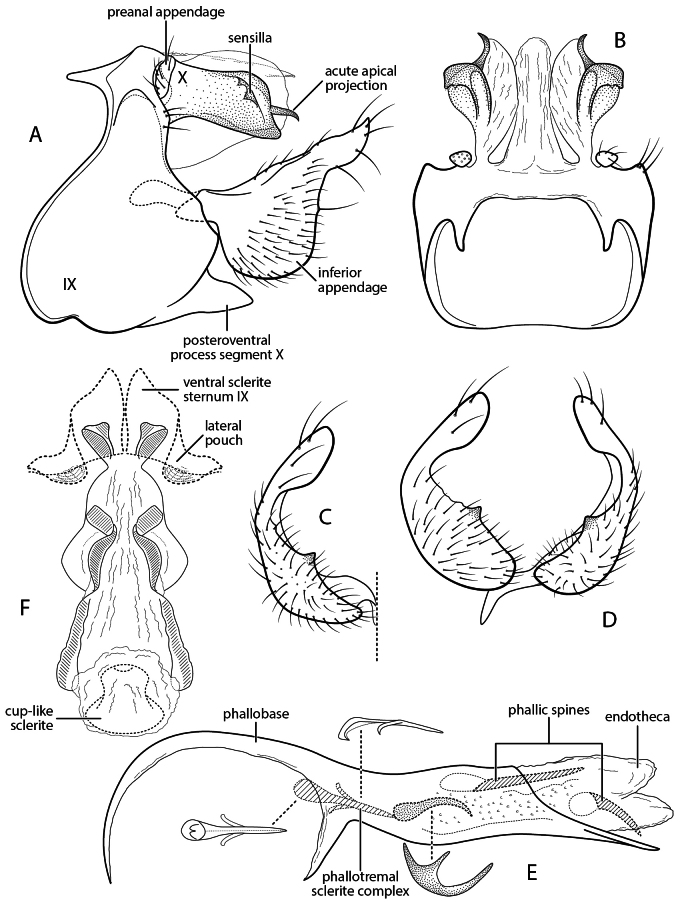
Chimarra (Chimarra) buenaventura new species. Male genitalia **A** segments IX, X, lateral **B** segments IX, X, dorsal **C** inferior appendage, ventral **D** inferior appendages, oblique lateral **E** phallus, lateral (insets: phallic spine, phallotremal sclerite) **F** female genitalia, vaginal apparatus, ventral.

###### Description.

**Adult.** Forewing length male 5.0 mm, female 5.0–5.25 mm (*n* = 3). Color pale fuscous, partially denuded (specimens in alcohol). **Male.** Abdominal segment IX, in lateral view, with very pronounced sinuous extension of anteroventral margin and short apodemes from anterodorsal margin; posteroventral process elongate, posteriorly projecting, subacute apically. Tergum X membranous mesally, with short, sclerotized lateral lobes; in lateral view, each more or less parallel-sided, subtruncate apically, with very narrow, acute, apical projection, projection laterally recurved, continuous with membranous dorsal extension of lateral lobe; sensilla of each lobe two, on reflexed sclerotized curl, near apex of sclerotized lateral lobe. Preanal appendage short, rounded, in lateral view, wider than long. Inferior appendage, in lateral view, with ventral margin broadly rounded; bearing minute triangular cusp on mesal margin, in ventral or caudal view; dorsal thumb-like process of inferior appendage, in lateral view, projecting posteriorly, strongly mesally curved, with apex expanded and rounded. Phallus with ventral margin of phallobase distinctly projecting; phallic spines two, asymmetrically arrayed, anterior spine elongate, straight, posterior spine shorter, slightly curved; endotheca textured with small spines and also possessing short, curved spine, with basal tine-like projection; phallotremal sclerite complex composed of elongate rod and ring structure and membranous structure with pair of associated wishbone-like sclerites apically; endotheca not expanded in specimen examined, but phallotremal sclerite complex apparently associated with short, curved endothecal spine.

**Female.** Ventral sclerites of sternum IX with prominent membranous lateral pouches (“clasper receptacles” of Blahnik 1998). Vaginal apparatus moderately elongate, largely membranous, with pair of distinctly cupped sclerites posteroventrally (attachment or anchoring site of vaginal apparatus), posterodorsal sclerites absent; at midlength, with rounded, membranous lateral projections, ventral surface with paired sclerites, narrow and sinuous, extending from membranous lateral projections to anterior cup-like sclerite, flanking lateral margin of vaginal apparatus anteriorly.

###### Etymology.

Named after Reserva Buenaventura of Fundación Jocotoco, located in the southwestern foothills of the Andes and contains elements of the Tumbesian dry forests of southern Ecuador and the Chocó humid forests of northwestern Ecuador. In addition to rare and endemic birds and plants, it is the type locality of this new species.

###### Remarks.

*Chimarra buenaventura* is a new species in the *ortiziana* group, as defined by Blahnik (1998). Additional species were subsequently described by Blahnik and Holzenthal (2012) and Holzenthal et al. (2022). The group currently contains fifteen species, distributed from Mexico to the northern parts of South America, with one species extending to southeastern Brazil. This new species adds a sixteenth species. Species of the group have a characteristically formed inferior appendage, with a relatively short and mesally curved, thumb-like, dorsal projection, usually somewhat dilated and rounded apically. The various species are most readily distinguished by the form and shape of the lateral lobes of tergum X, and the position and disposition of its two sensilla.

#### ﻿Philopotaminae

***Sumacodella***

The genus *Sumacodella* was only recently established for a monotypic species, *Sumacodella elongata* Holzenthal, Blahnik & Ríos-Touma, 2022, from Ecuador (Holzenthal et al. 2022). The new species described here adds a second species to the genus, also from Ecuador.

##### ﻿ Sumacodella grijalvai sp. nov.

Taxon classificationAnimaliaTrichopteraPhilopotamidae

3F639C6B-D673-5125-A476-885FA08D33B2

https://zoobank.org/FEE0D0A0-C26A-4F97-BE68-6860CFF40C19

Fig. 4

###### Type material.

***Holotype* male: Ecuador**: • **Imbabura**: Bosque Protector & Reser­va Biológica Los Cedros, 0.30966°N, 78,78196°W, el. 1400 m, 02.ix.2023, Ríos-Touma (UMSP000551804) (MECN).

###### Diagnosis.

Although morphologically distinct from *Sumacodella elongata*, this new species possesses almost all the apomorphic characters considered to be potentially diagnostic for the genus when it was established. Character similarities include a very elongate segment IX, with the posteroventral margin greatly produced, a dorsal margin that has almost disappeared, and a posterior margin from which both elongate, narrow preanal appendage and an extremely elongate and narrow, parallel-sided, tergum X emerge. The species are also similar in that the sensilla of tergum X are confined to a relatively short, digitate apicomesal projection, bordered laterally by narrow lateral projections. Both species also have inferior appendage with an extremely elongate anteromesal apodeme, and a very elongate and narrowly tubular phallus, lacking a basodorsal expansion. *Sumacodella grijalvai* differs from *S. elongata* in having segment IX even more elongate, with its anterolateral apodemes broader, and in having a tergum X that is also more elongate, almost excessively so, extending almost to the apex of the apical segment of the inferior appendage, with the lateral projections near its apex very narrow and laterally hooked apically, rather than simple and digitate. It lacks the short, apically setose projections found at the juncture of the mesal and lateral lobes of tergum X in *S. elongata*. The tubular phallus has an elongate, narrow, posteriorly directed, spine-like projection at approximately midlength, not present in *S. elongata*, and has only an indistinct tract of very small spines. Also, the apical segment of the inferior appendage is more broadly rounded apically, especially in lateral view.

**Figure 4.**
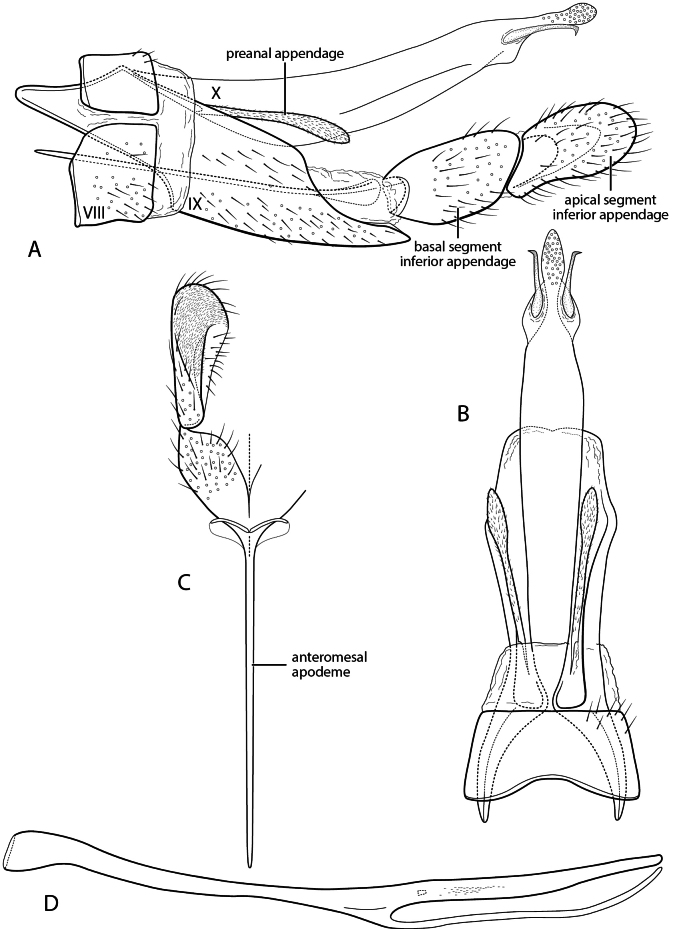
*Sumacodella grijalvai* new species. Male genitalia **A** segments VIII-X, lateral **B** segments VIII-X, dorsal **C** inferior appendage, dorsal **D** phallus, lateral.

###### Description.

**Adult.** Forewing length male 5.5 mm. Color pale brown, partially denuded (in alcohol). **Male.** Segment VIII short, sternum and tergum subequal in length, sternum densely covered with short, fine setae, tergum with setae confined to posterior region of segment, posterior margin with membranous extension, tightly surrounding segment IX. Segment IX, in lateral view, very elongate, strongly tapering, anterolateral margins modified dorsally to form pair of large, tapering apodemes, rounded apically, ventral margin strongly produced posteriorly, subtruncate in dorsal and ventral views, posterior margin very obliquely narrowed dorsally, with lateral margin converging from ca. mid-height to anterior margin; in dorsal view, with posterior margin forming V-shaped convergence at anterior margin. Tergum X extremely elongate, narrow, nearly parallel-sided, extending to (or almost to) apex of apical segment of inferior appendage; in lateral view, upturned in apical extremity forming digitate mesal lobe, densely covered with sensilla, bordered by pair of very narrow, sclerotized lateral lobes, shorter than mesal lobe, acute, laterally hooked apically. Preanal appendage elongate, extending nearly half length of tergum X, very narrow for most of length, widened preapically. Inferior appendage bi-segmented, segments subequal in length, nearly uniform in width; apical segment rounded, with dense pad of short, stiff apical spines, extended anteriorly on ventromesal surface; with extremely elongate anteroventral apodeme. Phallus very elongate, narrow, tubular, without basodorsal projection; ventrally with elongate, narrow, spine-like projection just past midlength, extending parallel to phallobase to its apex; phallus internally with only small tract of very minute spines, scarcely evident. Phallotremal sclerite very indistinct, weakly sclerotized, small, ring-like.

**Female.** Unknown.

###### Etymology.

We dedicate this species to Dr. Agustín Grijalva, a former Constitutional Judge of Ecuador, who successfully applied the constitutional “Rights of Nature” provision in a ruling that saved Bosque Protector Los Cedros from mining concessions. This Constitutional Court mandate protected this species and all those inhabiting Los Cedros.

#### ﻿*Wormaldia*

Neotropical species of the genus *Wormadia* were revised by Muñoz-Quesada and Holzenthal (2015). The revision recognized 50 species occurring in the region. Only three new species have been described since then: *W. imbrialis* Holzenthal, Blahnik & Ríos-Touma, 2018, from Ecuador; *W. noveloi* Razo-Gonzalez, 2018, from Mexico; and *W. natalis* Holzenthal, Blahnik & Ríos-Touma, 2022, from Ecuador. However, it is likely that several additional species from the region will eventually be recognized. The differences between species are often subtle. Characters of note are found in the shape and length of the inferior appendage, especially the form and arrangement of the modified setae on the apicomesal surface of the apical segment; modification on the posterior margin of tergum VIII; the form of tergum X, particularly its apex and modifications of the lateral margins; the length and shape of the spines of the phallus; and the general shape of segment IX, in lateral view. Modifications of the preanal appendage and posteromesal margin of sternites anterior to segment IX are also found in some species. Six species are currently known from Ecuador. The following three new species increase that number to nine.

##### ﻿ Wormaldia nilssoni sp. nov.

Taxon classificationAnimaliaTrichopteraPhilopotamidae

15DF0E28-6142-5A76-96CD-5B97CF92D35F

https://zoobank.org/FF344B67-7F70-4F95-A17C-0CA58F0E47B1

Fig. 5

###### Type material.

***Holotype* male: Ecuador**: • **Napo**: Wildsumaco Lodge, small stream #2, Benavides Trail, 0.67631°S, 77.59824°W, el. 1479 m, 13.xi.2023, Ríos, Holzenthal, Frandsen, Amigo (UMSP000551141) (MECN).

###### Diagnosis.

*Wormaldia nilssoni* most closely resembles *W. machadorum* Muñoz-Quesada & Holzenthal, 2015, described from Costa Rica and known also from Panama (Armitage et al. 2015). Similarities include the form of the inferior appendage, with a bulbous basal segment and apically narrowed apical segment, with a longitudinal arrangement of short spine-like setae on its mesal surface; and the narrow and lightly sclerotized posteromesal processes of tergum VIII. As compared to *W. machadorum*, the projections from tergum VIII are more V-shaped, closely situated basally and diverging apically. The anterior margin of segment IX is not as distinctly angular as it is in *W. machadorum*, or in other species mentioned as being close to that species in its original description. Other diagnostic features of *W. nilssoni* include the form of tergum X, with its apex slightly upturned and lateral margins broadly rounded, apparently with several scattered sensilla, and with a distinct short basomesal protuberance; and its phallic spines, one very short and curved, and the other lacking any noticeable projection (possibly as an aberration).

**Figure 5.**
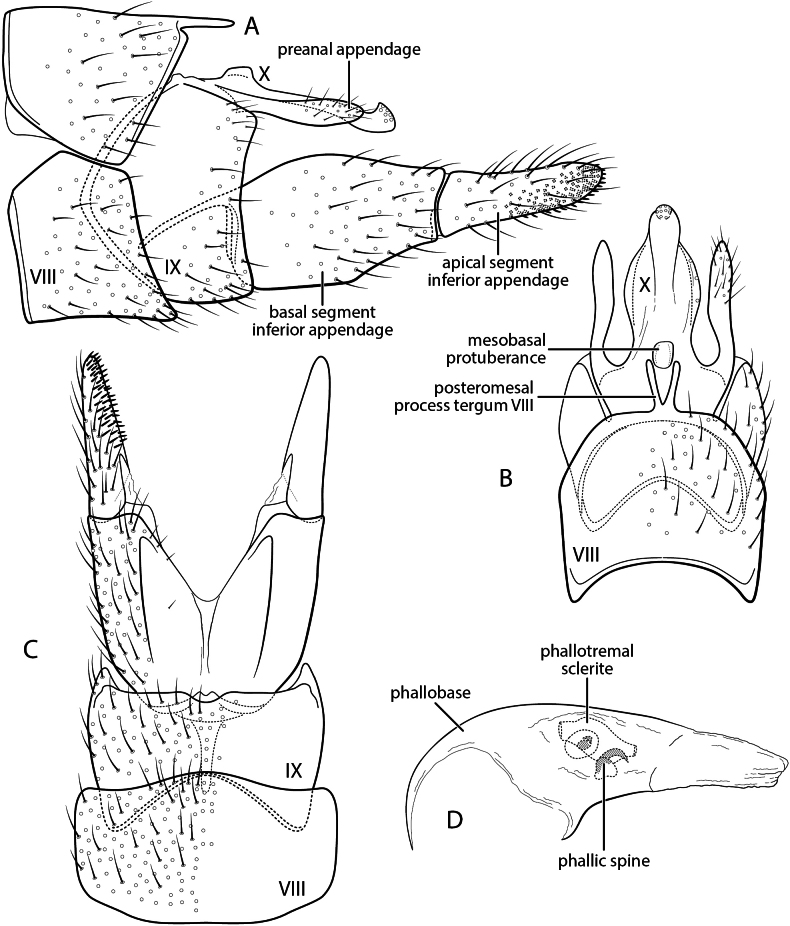
*Wormaldia nilssoni* new species. Male genitalia **A** segments VIII-X, lateral **B** segments VIII-X, dorsal **C** segments VIII-IX, ventral **D** phallus, lateral.

###### Description.

**Adult.** Forewing length male 4.5 mm. Color pale stramineous, denuded (specimen in alcohol). **Male.** Sternum VII without distinct posteromesal projection or process, very slightly convexly produced mesally. Tergum VIII with pair of posteromesal processes; processes weakly sclerotized, elongate, narrow, digitate, closely opposed basally, diverging apically, forming V-shaped configuration; sternum VIII unmodified, nearly straight. Segment IX, in lateral view, with anterolateral margin convexly rounded, very slightly angular in middle; posterior margin nearly straight (very slightly concave); dorsal margin abruptly narrowed from posterior margin, forming cleft extending almost to anterior margin, where continuous with tergum X and preanal appendage. Tergum X with apex sensillate, slightly enlarged and upturned, with small, subquadrate mesobasal protuberance; in dorsal view, with apex forming rounded projection, somewhat continuous anteromesally, lateral margins forming uniformly convex, but rather weakly projecting, lateral processes with several marginal sensilla. Preanal appendage apparently fused to base of tergum X basolaterally, simple in form, elongate, digitate, narrowed basally, shorter in length than tergum X. Inferior appendage with basal segment relatively elongate, bulbously enlarged at middle, narrowing apically; apical segment, in lateral view, distinctly shorter than basal segment, narrow, nearly uniform in width, narrowing apically; in ventral view, somewhat flattened on mesal surface, with patch of short, spine-like setae, extending longitudinally from apex. Phallus semimembranous, phallobase with bulbous basodorsal expansion, strongly narrowed apically, internally with very short, curved spine with enlarged base, and second basal small, sclerotized structure, lacking accompanying spine (possibly as aberration); phallotremal sclerite indistinct, apparently tubular, inflated at middle.

**Female.** Unknown.

###### Etymology.

This species is dedicated to Jonas Nilsson, founder of Wildsumaco Lodge and its surrounding preserved forest, for his efforts to protect this amazing habitat and its near pristine forest.

##### ﻿ Wormaldia insolita sp. nov.

Taxon classificationAnimaliaTrichopteraPhilopotamidae

6402FC81-EE16-59FE-9B41-2C722C32BA25

https://zoobank.org/20529AB3-5978-446B-8F22-EF659C79A859

Fig. 6

###### Type material.

***Holotype* male: Ecuador**: • **Pichincha**: small forest stream, Milpe Bird Sanctuary, 0.03249°N, 78.86681°W, el. 1125 m, 20.xi.2023, Ríos, Holzenthal, Frandsen, Amigo (UMSP000507032) (UMSP). ***Paratype*: Ecuador**: • **Pichincha**: small trickle, Milpe Garden, Mindo Cloud Forest Foundation, 0.03739°N, 78.87086°W, el. 1114 m, 21.xi.2023, Ríos, Holzenthal, Frandsen, Amigo, 1 male (UMSP000551499) (MECN).

###### Diagnosis.

*Wormaldia insolita*, because of the very distinctive and unusual development of its inferior appendage, is readily identified and unlikely to be confused with any other described species. Particularly diagnostic is the apical segment of the inferior appendage, which has its apex strongly deflexed and narrowed, and has a longitudinal tract of very dense and minute, spine-like setae on its mesal surface, not quite extending to the apex. The basal segment of the inferior appendage is very bulbously enlarged. Like *W. milpe* sp. nov. and related species, it has a distinctive mesal notch on the posterior margin of tergum VIII. However, we are uncertain about the closest affinity of this new species.

###### Description.

**Adult.** Forewing length male 4.5 mm (*n* = 2). Color fuscous. **Male.** Sternum VII without distinct posteromesal projection or process, very slightly convexly produced mesally. Tergum VIII with posterior margin modified to form broad mesal notch, bordered laterally by short rounded sclerotized projections, margin of notch also sclerotized; sternum VIII unmodified, only weakly notched mesally. Segment IX, in lateral view, with anterolateral margin broadly and convexly rounded, posterior margin nearly straight, narrowing dorsally. Tergum X, in lateral view, with apex rounded, sensillate; preapically, on dorsal margin, with concavely rounded excavation; tergum, as viewed dorsally, subtriangular, narrow apically, widened basally, with small, subtriangular lateral projections at approximately midlength. Preanal appendage elongate, digitate, somewhat irregular in shape, extending to preapical excavation of tergum X, apparently fused to posterior margin of segment IX, lateral to base of tergum X. Inferior appendage, in lateral view, with basal segment short and very wide, length subequal to width, wide at apex; apical segment, in lateral view, subequal in length to basal segment, basally as wide as apex of basal segment, extending short distance and then abruptly ventrally flexed from dorsal margin, narrowed, acute apically, with apex projecting downward; posterior and apical margins of apical segment with elongate setae, pad of very dense, minute, spine-like setae on mesal surface, though not exposed in lateral view, distinctly visible along posterior margin, visible in dorsal view, but not directly visible, though apparent, in ventral view; in ventral view, both segments of inferior appendage much narrower and more uniform in width, basal segment wider than apical segment, apical segment subacute apically. Phallus with phallobase expanded basodorsally, very short ventrally, sclerotization extending laterally into endotheca; endotheca moderately elongate, with two elongate, narrow spines, one very strongly curved, with enlarged base, other very narrow and sinuous, with base narrow and nearly as elongate as spine. Phallotremal sclerite indistinct, apparently widened or flared apically.

**Figure 6.**
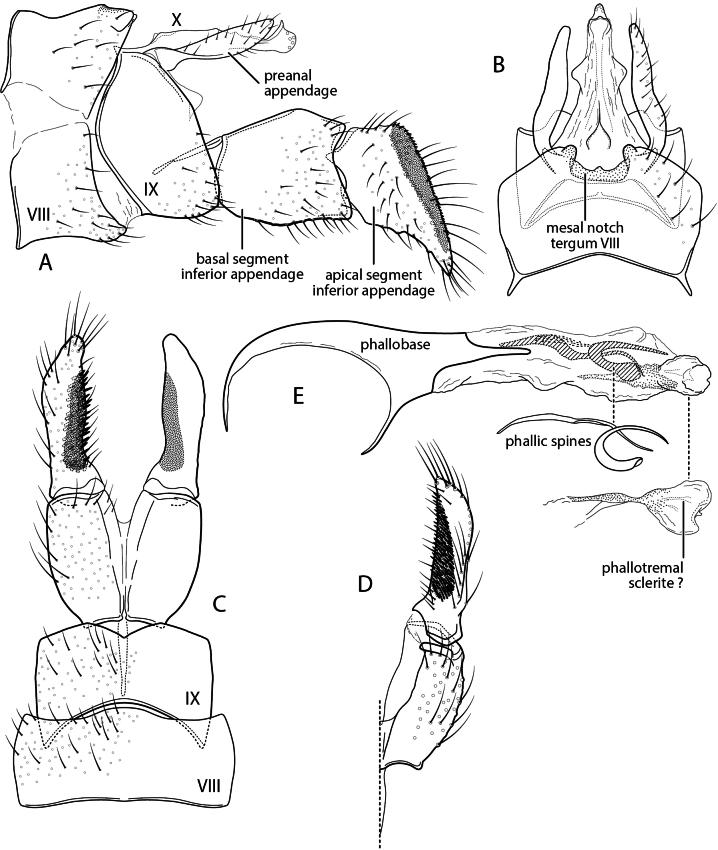
*Wormaldia insolita* new species. Male genitalia **A** segments VIII-X, lateral **B** segments VIII-X, dorsal **C s**egments VIII-IX, ventral **D** inferior appendage, dorsal **E** phallus, lateral (insets: phallic spines, phallic apex, oblique).

**Female.** Unknown.

###### Etymology.

From the Latin *insolita* meaning unusual, uncommon or strange, and attributed to the unusual development of its inferior appendage, which are unique for this species in the genus.

##### ﻿ Wormaldia milpe sp. nov.

Taxon classificationAnimaliaTrichopteraPhilopotamidae

AB19308D-3163-58CF-A5E3-5067DCEC1741

https://zoobank.org/F3C9C15C-BBA9-4E25-952E-9F748106CE7A

Fig. 7

###### Type material.

***Holotype* male: Ecuador: Pichincha**: small trickle, Milpe Garden, Mindo Cloud Forest Foundation, 0.03739°N, 78.87086°W, el. 1114 m, 21.xi.2023, Ríos, Holzenthal, Frandsen, Amigo (UMSP000551500) (MECN).

###### Diagnosis.

*Wormaldia milpe* most closely resembles *W. gallardoi* Muñoz-Quesada & Holzenthal, 2015, and several related species, including *W. andrea*, Muñoz-Quesada & Holzenthal, 2015; *W. prolixa* Flint, 1991; and *W. francovilla* Muñoz-Quesada & Holzenthal, 2015. These species have the apical segment of the inferior appendage approximately subequal in length to the basal segment, only moderately narrower in width, with the apex slightly enlarged in lateral view, and with the apicomesal spine-like setae somewhat longitudinally arranged. The species also have a similar posteromesal notch on tergum VIII, though variably developed, and a tergum X with a distinctly enlarged apex and modified lateral margins. Distributions of the species range from Costa Rica through Colombia. *Wormaldia milpe* has the posteromesal notch of tergum VIII relatively wide and shallow, with short rounded lateral projections and the mesal notch bordered posteriorly with short angular projections. Tergum X has an apex that is sensillate and very sharply upturned; basally it has a distinctly rounded and flattened projection, with its posterior margin slightly projecting; the lateral margins of tergum X have a uniformly rounded lateral projection in the basal half, with several scattered sensilla. The phallic spines are very unequal in size, one very short and curved, the other much longer and slightly curved; both spines have their bases enlarged, that of the longer spine forming an elongate sclerotized curl. The above characters, considered collectively, adequately diagnosis this new species.

**Figure 7.**
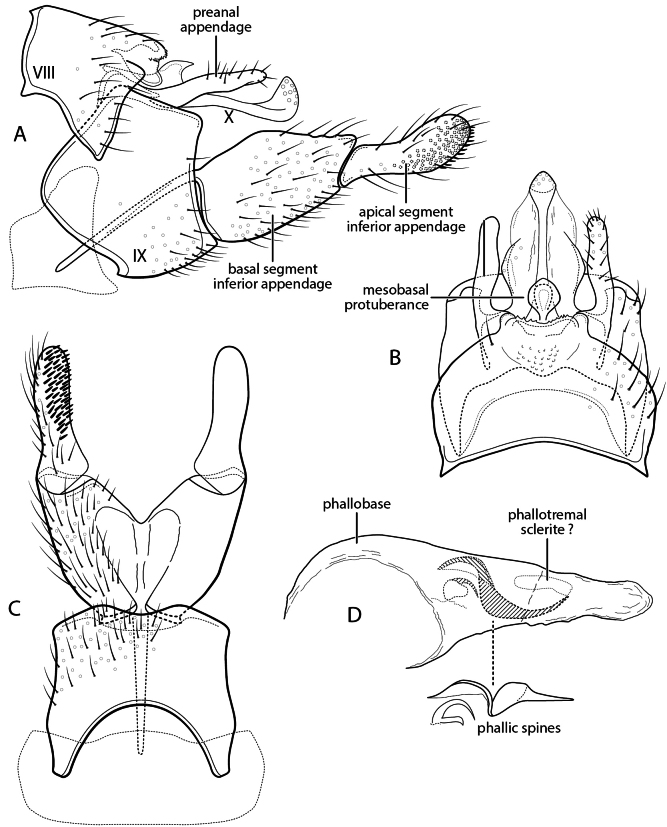
*Wormaldia milpe* new species. Male genitalia **A** segments VIII-X, lateral **B** segments VIII-X, dorsal **C s**egments VIII-IX, ventral **D** phallus, lateral (inset: phallic spines).

###### Description.

**Adult.** Forewing length male 3.5 mm. Color pale stramineous, denuded (specimen in alcohol). **Male.** Sternum VII without distinct posteromesal projection or process, very slightly convexly produced mesally. Tergum VIII with posterior margin modified mesally to form broad and shallow notch, bordered laterally by short, rounded projections, margin of notch with acute sclerotized projections; sternum VIII unmodified, only shallowly indentate mesally. Segment IX, in lateral view, with anterolateral margin convexly rounded, very slightly angular in middle; posterior margin nearly straight (very slightly concave); dorsal margin abruptly narrowed from posterior margin, forming cleft extending almost to anterior margin, where continuous with tergum X and preanal appendage. Tergum X, in lateral view, with apex sensillate, distinctly upturned and enlarged, with dorsally flattened mesobasal protuberance, with posterior margin distinctly projecting; mesobasal protuberance, in dorsal view, rounded, with narrowed base; tergum X, in dorsal view, with apex rounded, narrowing to form continuous narrow mesal sclerotization, lateral margins more membranous, widened anterior to sensillate mesal lobe, subparallel, in basal half forming uniformly convex, but weakly projecting, lateral processes with several marginal sensilla. Preanal appendage apparently fused to base of tergum X basolaterally, digitate, slightly irregular in shape, distinctly shorter in length than tergum X. Inferior appendage with basal segment wide, slightly elongate, enlarged at middle, narrowing apically; apical segment, in lateral view, slightly shorter than basal segment, narrower basally than apex of basal segment, slightly widened and rounded apically; in ventral view, with apex rounded, with tract of short, spine-like setae, extending longitudinally from apex. Phallus semi-membranous, phallobase with bulbous basodorsal expansion, strongly narrowed apically, internally with very short, curved spine with enlarged base, and second much longer, curved, apically acute spine, with elongate, curled basal sclerotization; phallotremal sclerite indistinct, apparently short and tubular.

**Female.** Unknown.

###### Etymology.

Named after the type locality, the town of Milpe and the nearby preserved cloud forest, managed by the laudable work of the Mindo Cloud Forest Foundation.

### ﻿Diversity and distribution of the Philopotamidae of Ecuador

**Figure 8.**
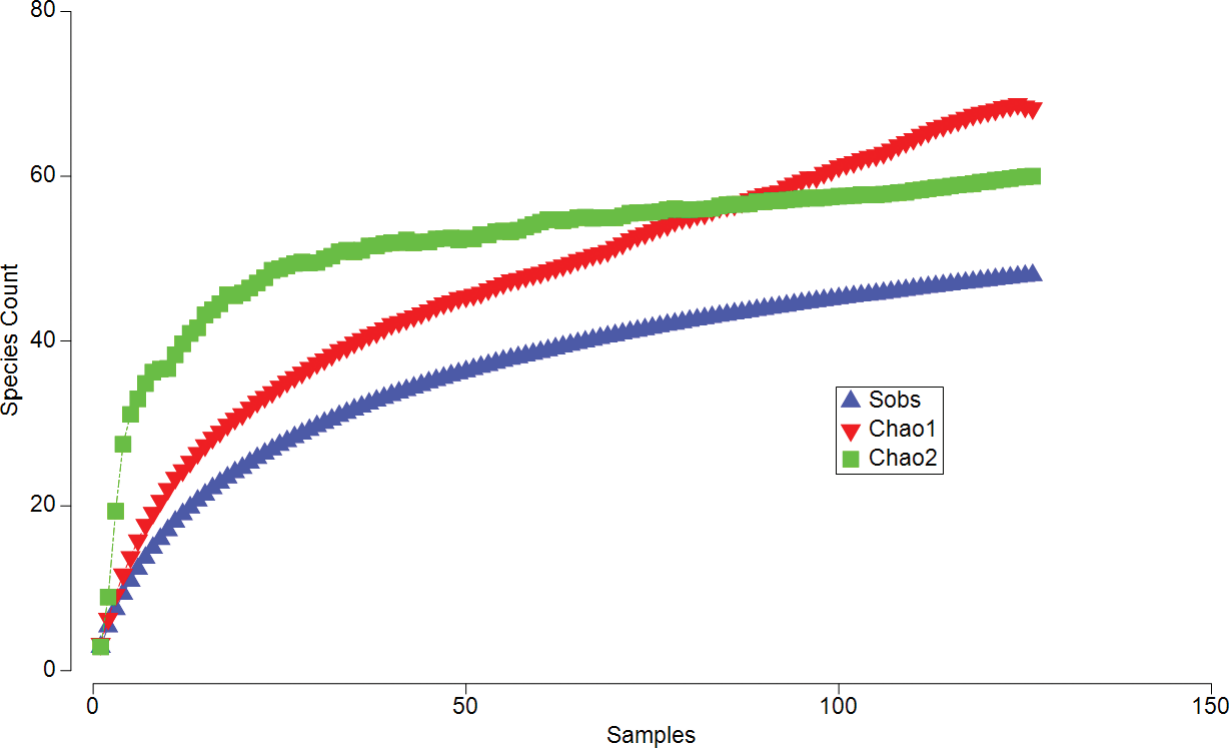
Species accumulation plot for Philopotamidae species in Ecuador, based on our collecting efforts. Species observed (Sobs) are in blue; Chao 1 species estimator (in red, abundance-based), and Chao 2 species estimator (in green, incidence-based) curves are also shown in the figure. Chao 2 predicts that species observed (Sobs) are close to the potential species richness.

With the currently described species, Ecuador has 60 Philopotamidae species, 36 of which are endemic. We have collected 48 of these 60 species during our collecting efforts. The Chao 2 estimator, based on 126 localities, suggests that the expected number of species is 60, but with an SD of 9 (Fig. 8, Suppl. material 1: table S1), which implies that almost all the philopotamid species in the country are known. However, 11 species from our collections are only known from one or two individuals, which means that rare species are quite common, and more of these species may appear with further collecting efforts. Localities ranged from 1 to 9 species of Philopotamidae per site. The most diverse genus is *Chimarra*, with 41 species, followed by *Wormaldia*, with ten species (Table 1). *Sumacodella*, a recently described genus, is known from two rare new species. The family is widely distributed across the country, on both sides of the Andean Ranges, from 50 to 4,200 meters above sea level (m asl). However, the middle elevations (800 to 1,800 m asl) are the richest in species.

**Table 1.** Species of Philopotamidae known for Ecuador.

Species	Known locality, by province	Endemic	Elevation range (meters)
*** Chimarra ***			
***Chimarra*** subgenus			
*C. asterae* Holzenthal, Blahnik & Ríos-Touma, 2022	Napo, Morona Santiago	E	551–1170
* C. * ***buenaventura* sp. nov.**	El Oro	E	530
*C. coheni* Blahnik, 1998	Pichincha	E	335
*C. creagra* Flint, 1981	Morona Santiago, Napo	E	1076
*C. decimlobata* Flint, 1991	Imbabura		1312–1587
*C. dolabrifera* Flint & Reyes, 1991	Pichincha, Cotopaxi, Esmeraldas, Los Ríos		335
*C. duckworthi* Flint, 1967	Pastaza		literature record, elevation not provided
*C. emima* Ross, 1959	Pichincha, Cotopaxi, Loja, Los Ríos, Santo Domingo		220–550
*C. inflata* Blahnik, 1998	Napo (Sucumbíos)	E	300
*C. langleyae* Blahnik, 1998	Napo (Sucumbíos)	E	300
*C. longiterga* Blahnik & Holzenthal, 1992b	Manabí, Pichincha (Santo Domingo)		220
*C. mashpi* Holzenthal, Blahnik & Ríos-Touma, 2022	Pichincha, Cotopaxi	E	473–1125
*C. munozi* Blahnik & Holzenthal, 1992b	Pichincha		570–700
*C. onima* Flint, 1991	Pichincha, Santo Domingo		700
*C. pacifica* Holzenthal, Blahnik & Ríos-Touma, 2022	El Oro, Pichincha	E	498–1238
*C. paracreagra* Blahnik, 1998	Pastaza, Morona Santiago, Tungurahua	E	1280–1531
*C. peineta* Blahnik & Holzenthal, 1992b	Los Ríos, Santo Domingo, Pichincha		220–550
*C. pumila* (Banks, 1920)	Los Ríos	E	50
*C. quadratiterga* Blahnik, 1998	Zamora-Chinchipe	E	980–1340
*C. rafita* Blahnik, 1998	Pastaza	E	1000
*C. strongyla* Blahnik, 1998	Pichincha	E	1100
*C. utra* Blahnik, 1998	Pastaza, Morona Santiago	E	1076
*C. xus* Blahnik, 1998	Pastaza, Napo, Pichincha, El Oro		570–1456
*C. zamora* Blahnik, 1998	Zamora-Chinchipe,		980
***Chimarrita*** subgenus			
*C. prolata* Blahnik, 1997	Pastaza	E	970
***Curgia*** subgenus			
*C. acinaciformis* Flint, 1998	Pastaza, Napo, Morona Santiago	E	1067–1479
*C. amigo* Holzenthal, Blahnik & Ríos-Touma, 2022	Carchi, Pichincha	E	1258–1669
*C. centralis* Ross, 1959	Pichincha		700
*C. didyma* Flint, 1998	Pichincha, Cotopaxi		335–1100
*C. geranoides* Flint, 1998	Pichincha, Pastaza, Tungurahua, Zamora Chinchipe		980–4200
*C. immaculata* Ulmer, 1911	Napo, Pastaza, Sucumbíos, Orellana		498–706
*C. lojaensis* Flint, 1998	Zamora-Chinchipe	E	2000
*C. macara* Flint, 1998	Loja	E	650
*C. margaritae* Flint, 1991	Tungurahua		1550
*C. otuzcoensis* Flint & Reyes, 1991	Pichincha		2000
*C. pablito* Flint, 1998	Pichincha		570
*C. persimilis* Banks, 1920	Los Ríos, Esmeraldas, Pichincha, Loja, Cotopaxi, Manabí		225–600
*C. peruviana* Flint, 1998	Napo, Pastaza		950
*C. puya* Flint, 1998	Pastaza	E	literature record, elevation not provided
***Otarrha*** subgenus			
***C. buglas* sp. nov.**	Morona Santiago	E	1016
*C. ramosa* Holzenthal, Blahnik & Ríos-Touma, 2022	Orellana, Pastaza	E	610–703
**unplaced to subgenus***C. usitatissima* Flint, 1971	Sucumbíos		230–250
*** Chimarrhodella ***			
*C. aequatoria* (Navás, 1934)	Loja	E	literature record, elevation not provided
*C. choco* Holzenthal, Blahnik & Ríos-Touma, 2018	Carchi, Bolivar, Imbabura, Pichincha	E	850–1660
*C. ornata* Blahnik, 2004	Tungurahua	E	1600
*C. peruviana* (Ross, 1956)	Morona Santiago, Sucumbíos		1416 –1777
**C.*spinosa* sp. nov.**	Zamora Chinchipe	E	1898
*C. ulmeri* (Ross, 1956)	Morona Santiago, Pastaza, Tungurahua	E	1076–1280
*** Hydrobiosella ***			
*H. andina* Holzenthal, Blahnik & Ríos-Touma, 2018	Tungurahua	E	1600
*** Sumacodella ***			
*S. elongata* Holzenthal, Blahnik & Ríos-Touma, 2022	Napo	E	1456
***S. grijalvai* sp. nov.**	Imbabura	E	1400
*** Wormaldia ***			
*W. andrea* Muñoz-Quesada & Holzenthal, 2015	Tungurahua	E	1550
*W. araujoi* Muñoz-Quesada & Holzenthal, 2015	Napo	E	640
*W. imbrialis* Holzenthal, Blahnik & Ríos-Touma, 2018	Pichincha	E	1111–1180
***W. insolita* sp. nov.**	Pichincha	E	1114 –1125
***W. milpe* sp. nov.**	Pichincha	E	1114
*W. natalis* Holzenthal, Blahnik & Ríos-Touma, 2022	Napo	E	1420
***W. nilssoni* sp. nov.**	Napo	E	1479
*W. planae* Ross & King, in Ross, 1956	Los Ríos, Pichincha, Santo Domingo		250 –1250
*W. sumaco* Holzenthal, Blahnik & Ríos-Touma, 2022	Napo	E	1456

## ﻿Discussion

Since 2017, 17 species, one continental genus record, and one new genus of Philopotamidae have been described for Ecuador (Ríos-Touma et al. 2017; Holzenthal et al. 2018, 2022). Of these, at least nine are known from a single male specimen. This highlights the importance of rare species in Trichoptera and other aquatic insects (Ríos-Touma et al. 2023). Based on incidence data, the Chao 2 species richness estimator suggests that we are close to finding the expected number of species in the country. However, given that only a few or a single specimen collected is known for more than 30% of the species, we would expect more rare species yet to be discovered.

At the same time, some species seem to be abundant and widely distributed (Suppl. material 1: table S1): *Chimarra creagra*, *Chimarra decimolobata*, *Chimarra emima*, *Chimarra onima*, *Wormaldia andrea* and *Wormandia planae* (Table 1) are very widely distributed in the country and can be locally abundant. The family is very diverse in middle elevations (1000 to 2000 m a.s.l.). The only species we have collected above 2000 m a.s.l. is *Chimarra geranoides*. Meanwhile, *Chimarrhodella*, *Sumacodella*, and *Wormaldia* seem to be restricted to middle elevations (Table 1). Moreover, at the order level, these middle elevations seem to be highly diverse, with high beta diversity for species and genera of Trichoptera (Ríos-Touma et al. 2022). Therefore, more efforts are needed to collect in these areas, where more species are expected to exist. This is also important, facing the increasing pressure due to mining concessions and other negative impacts on the ecosystems (Roy et al. 2018).

## Supplementary Material

XML Treatment for Chimarrhodella spinosa

XML Treatment for Chimarra (Otarrha) buglas

XML Treatment for Chimarra (Chimarra) buenaventura

XML Treatment for Sumacodella grijalvai

XML Treatment for Wormaldia nilssoni

XML Treatment for Wormaldia insolita

XML Treatment for Wormaldia milpe

## ﻿Additional information

### Author ORCIDs

Ralph W. Holzenthal https://orcid.org/0000-0003-1853-6340

Roger J. Blanhik https://orcid.org/0000-0002-0648-454X

Blanca Ríos-Touma https://orcid.org/0000-0002-3921-0908

### Data availability

All of the data that support the findings of this study are available in the main text or Supplementary Information.

## Supplementary materials

Supplementary material 1 Observed species and species estimator Chao 2 for all 126 localities sampled in Ecuador Data type xlsx https://binary.pensoft.net/file/1488379This dataset is made available under the Open Database License (http://opendatacommons.org/licenses/odbl/1.0/). The Open Database License (ODbL) is a license agreement intended to allow users to freely share, modify, and use this Dataset while maintaining this same freedom for others, provided that the original source and author(s) are credited.Ralph W. Holzenthal, Roger J. Blanhik, Blanca Ríos-Touma
